# Supplementary material for: P-TRAP: a Panicle Trait Phenotyping tool
Source: BMC Plant Biol. 2013 Aug 29;13:122. doi: 10.1186/1471-2229-13-122 (PMC3848748; doi:10.1186/1471-2229-13-122)
Supplement: Additional file 7 — P-TRAP source code. The Netbeans project that contains the source code of the application. [file 1471-2229-13-122-S7.zip › souce.zip!/P-TRAP source code/Grain/doc/javadoc/org-basmati-fileexplorer/org/basmati/fileexplorer/package-frame.html]

org.basmati.fileexplorer (File Explorer)


# org.basmati.fileexplorer

## Classes

- RiceImageDataObject
